# Prognostic Score for *De Novo* Metastatic Breast Cancer With Liver Metastasis and Its Predictive Value of Locoregional Treatment Benefit

**DOI:** 10.3389/fonc.2021.651636

**Published:** 2021-08-27

**Authors:** Lei Ji, Lei Fan, Xiuzhi Zhu, Yu Gao, Zhonghua Wang

**Affiliations:** ^1^Department of Medical Oncology, Fudan University Shanghai Cancer Center, Shanghai Medical College, Fudan University, Shanghai, China; ^2^Department of Oncology, Shanghai Medical College, Fudan University, Shanghai, China; ^3^Department of Breast Surgery, Fudan University Shanghai Cancer Center, Shanghai Medical College, Fudan University, Shanghai, China

**Keywords:** breast cancer, liver metastasis, prognostic factors, prognostic model, risk stratification, predictive value

## Abstract

**Background:**

There is a significant survival difference and lack of effective treatment among breast cancer patients with liver metastasis. This present study aimed to construct a novel prognostic score for predicting the prognosis and locoregional treatment benefit of *de novo* metastatic breast cancer with liver metastasis (BCLM).

**Methods:**

In total, 2,398 eligible patients between 2010 and 2016 were selected from the Surveillance, Epidemiology, and End Results (SEER) database. They were assigned to the training set including 1,662 patients (2010–2014) and validation set comprising 736 patients (2015–2016) depending on the time of diagnosis. The prognostic score was based on regression coefficients in the multivariate Cox regression analysis. And then, patients were stratified into low-, intermediate-, and high-risk groups by the prognostic score. The discrimination and calibration of prognostic score were evaluated using time-dependent receiver operating characteristic (ROC) curves analysis and calibration curves, respectively. Subgroup analysis was performed to evaluate locoregional surgery and chemotherapy benefit in different risk groups.

**Results:**

Age, race, insurance and marital status, T stage, pathological grade, molecular subtypes, and extrahepatic metastasis were identified as independent prognostic variables in the prognostic score. The prognostic score showed high discrimination power with an area under the curve (AUC) of 0.77 and 0.72 and excellent agreement suggested by calibration plots in the training and validation sets, respectively. Intermediate-risk [hazard ratio (HR) 2.39, 95% confidence interval (CI) 2.09–2.73, *P*<0.001] and high-risk groups (HR 4.88; 95% CI 4.13–5.76; *P*<0.001) had significantly worse prognosis in comparison with the low-risk group. The median overall survival (OS) in three prognostic groups were 44, 18, and 7 months, with a 3-year survival rate of 56, 23, and 7%, respectively. Apart from the high-risk group (HR 0.79; 95% CI 0.56–1.10; *P*=0.157), the low-risk (HR 0.64; 95% CI 0.49–0.84; *P*=0.001) and intermediate-risk groups (HR 0.68; 95% CI 0.55–0.85; *P*=0.001) could benefit from the surgery of primary site, while chemotherapy improved prognosis in all risk groups.

**Conclusions:**

A prognostic score was developed to accurately predict the prognosis of *de novo* BCLM patients. Moreover, it may be useful for further subdividing them into different risk groups and helping guide clinicians in treatment decisions.

## Introduction

Together with bone, lung, and brain, the liver is a common target organ of distant metastasis among breast cancer patients, with liver metastasis as the first distant metastasis site occurring in nearly 30% of breast cancer patients (1–3). Despite advances in diagnosis and treatment of metastatic breast cancer, liver metastasis remains a major cause of cancer-related death in breast cancer patients. The overall survival of breast cancer with liver metastasis (BCLM) is generally short and only 3–6 months in untreated patients (4). However, BCLM is a heterogeneous disease with diverse clinical outcomes influenced by demographic or clinicopathological factors, including age, race, insurance and marital status, pathological grade, hormone receptor (HR) status, human epidermal growth factor receptor 2 (HER2) status, serum albumin level, extrahepatic metastasis, performance status, and treatment options (1–3). Previous studies reported a dramatically different prognosis with median overall survival ranging from 4 to 82 months in BCLM patients, indicating that BCLM is a heterogeneous disease state (1–3, 5). Several prognostic models based on clinical features or genetic biomarkers have been constructed to predict the outcome of metastatic breast cancer (MBC) or breast cancer with brain metastasis (BCBM), because the clinical behavior of MBC, including BCBM and BCLM, is characterized by heterogeneity (6–8). Unfortunately, there was no sensitive and specific predictive tool for BCLM at first presentation.

Presently, the National Comprehensive Cancer Network (NCCN) panel recommended systemic therapy as the primary treatment strategy for recurrent or stage IV breast cancer (9). However, it should be noted that BCLM seemed to be insensitive to traditional endocrine therapy and current immunotherapy because treatment response could vary extensively depending on metastatic sites (10, 11). As a result, there was a lack of effective treatment approach for BCLM patients unresponsive to systemic therapy. In recent years, a growing number of studies have suggested that local treatment of breast cancer liver metastases, such as hepatic metastasectomy, radiofrequency ablation (RFA), and transarterial chemoembolization (TACE), could confer encouraging survival benefit (5, 12). Meanwhile, some retrospective studies also indicated that complete excision of the primary tumor in selected patients with *de novo* metastatic breast cancer showed a potential survival benefit (13). Despite substantial selection biases and confounding results in these studies, locoregional treatment of primary tumor or liver metastases may offer an additional option for selected BCLM patients. Therefore, the objective of our study was to build a novel prognostic score for better prognosis prediction of *de novo* metastatic breast cancer with liver metastasis and help identify patients who can benefit from locoregional treatment of the primary tumor.

## Materials and Methods

**Figure 1:**
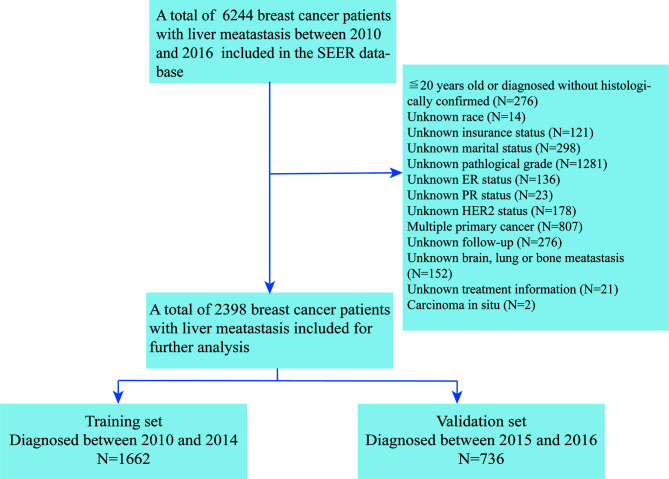
Selection of patients.

The present study used a retrospective cohort study design. *De novo* BCLM patients were defined as metastatic (stage IV) breast cancer patients with liver metastasis at initial diagnosis. Data of 6,244 *de novo* BCLM patients diagnosed between 2010 and 2016 were extracted from the Surveillance, Epidemiology, and End Results (SEER) database. Other inclusion and exclusion criteria for the study were given below. Inclusion criteria: (1) age >20 years old; (2) histologically confirmed invasive breast cancer; (3) complete demographic and clinicopathological data including race, insurance and marital status, pathological grade, T and N stage, molecular subtype, and treatment information. Exclusion criteria: (1) unknown extrahepatic metastasis; (2) multiple primary cancer; (3) unknown follow-up status. Ultimately, 2,398 *de novo* BCLM patients were included in this study for final analysis. The flowchart for selecting eligible patients is presented in the **Figure 1**.

Then, all the samples were split into a training set (n=1,662, 2010–2014) and a validation set (n=736, 2015–2016) based on the time of diagnosis. In the training set, multivariate Cox regression models were performed to identify independent prognostic factors without considering treatment options and estimate the hazard ratios (HRs) for these factors by backward stepwise procedure. In the Cox proportional hazards model, regression coefficients of these prognostic factors indicated the contribution of each factor to the overall survival (OS). Therefore, they could be converted to a prognostic score of each independent prognostic factor after being multiplied by 10 and rounded. Overall prognostic score, the sum of points of these factors, was used to evaluate death risk and predict outcome of *de novo* BCLM patients. Subsequently, the discrimination and calibration of the prognostic score were evaluated using time-dependent receiver operating characteristic (ROC) curves analysis and calibration curves, respectively. The area under the ROC curves (AUC) was calculated, and calibration curves were plotted in both the training set and validation set for internal or external validation. Furthermore, X-tile software was used to determine the optimal cut-off value of risk stratification according to overall prognostic score, making it possible to divide all patients into low-, intermediate-, and high-risk groups.

Correlation between baseline characteristics and different risk groups was also evaluated using a chi-squared test or Fisher’s exact test. The survival difference among different risk groups was compared by Kaplan-Meier method using log-rank test. Subgroup analysis was performed to explore locoregional surgery and chemotherapy benefit in different risk groups using separate Cox models containing all covariates in each individual subgroup. Forest plots were drawn to visualize these results. Two-sided P values < 0.05 represented statistical significance. Statistical analyses were executed with SPSS 26.0 (Chicago, IL, USA) and R software version 4.0.0.

## Results

### Baseline Characteristics and Its Relationship With Risk Stratification

In total, 2,398 *de novo* BCLM patients were included, of which 1,662 patients were in the training set and 306 patients were in the validation set. In the training set, *de novo* BCLM patients tended to be younger than 60 years old (59.3%), insured (96.0%) and unmarried (51.5%) white (74.1%) patients. In addition, most patients had relatively higher T staging (T3 or T4, 54.4%) but lower N staging (N0 or N1, 70.7%). The majority of these patients were identified as high-grade (III/IV, 60.5%) ductal carcinoma (83.9%). Of the 1,662 patients, 1,109 (66.7%) had hormonal receptors (HR) positive disease and 979 (58.9%) had human epidermal growth factor receptor2 (HER2) negative disease. *De novo* BCLM patients often had other combined metastases, including bone (57.4%), lung (33.5%), and brain metastasis (7.1%). The majority of patients received first-line chemotherapy (73.6%), while only few patients underwent surgery of primary site (33.4%) or radiotherapy (15.2%). Many demographic characteristics and clinicopathological features showed differences among different risk groups, indicating the potential prognostic value of these factors. Detailed baseline characteristics and its relationship with risk stratification are shown in **Table 1** and **Supplementary Table 1**.

**Table 1:** Characteristics of BCLM and risk stratification in the training set.

		Risk stratification			*P* Value
	Total N = 1,662 (100%)	Low-risk N = 716 (43.1%)	Intermediate-risk N = 676 (40.7%)	High-risk N = 270 (16.2%)	
Age at initial diagnosis, years					
<60	985 (59.3%)	572 (79.9%)	338 (50.0%)	75 (27.8%)	<0.001
≥60	677 (40.7%)	144 (20.1%)	338 (50.0%)	195 (72.2%)	
Gender					
Male	7 (0.4%)	3 (0.4%)	3 (0.4%)	1 (0.4%)	0.988
Female	1,655 (99.6%)	713 (99.6%)	673 (99.6%)	269 (99.6%)	
Race					
White	1,232 (74.1%)	564 (78.8%)	495 (73.2%)	173 (64.1%)	<0.001
Black	307 (18.5%)	85 (11.9%)	138 (20.4%)	84 (31.1%)	
Asian or PI	117 (7.0%)	64 (8.9%)	41 (6.1%)	12 (4.4%)	
AI or AN	6 (0.4%)	3 (0.4%)	2 (0.3%)	1 (0.4%)	
Insurance status					
Uninsured	66 (4.0%)	19 (2.7%)	22 (3.3%)	25 (9.3%)	<0.001
Insured	1,596 (96.0%)	697 (97.3%)	654 (96.7%)	245 (90.7%)	
Marital status					
Unmarried	856 (51.5%)	274 (38.3%)	386 (57.1%)	196 (72.6%)	<0.001
Married	806 (48.5%)	442 (61.7%)	290 (42.9%)	74 (27.4%)	
T					
1	183 (11.0%)	101 (14.1%)	61 (9.0%)	21 (7.8%)	<0.001
2	575 (34.6%)	311 (43.4%)	215 (31.8%)	49 (18.1%)	
3	312 (18.8%)	150 (20.9%)	132 (19.5%)	30 (11.1%)	
4	592 (35.6%)	154 (21.5%)	268 (39.6%)	170 (63.0%)	
N					
0 or 1	1,175 (70.7%)	510 (71.2%)	492 (72.8%)	173 (64.1%)	0.084
2	227 (13.7%)	93 (13.0%)	91 (13.5%)	43 (15.9%)	
3	260 (15.6%)	113 (15.8%)	93 (13.8%)	54 (20.0%)	
Histological type					
IDC	1,394 (83.9%)	603 (84.2%)	566 (83.7%)	225 (83.3%)	0.008
ILC	83 (5.0%)	29 (4.1%)	47 (7.0%)	7 (2.6%)	
Other	185 (11.1%)	84 (11.7%)	63 (9.3%)	38 (14.1%)	
Pathological grade					
I	69 (4.2%)	50 (7.0%)	18 (2.7%)	1 (0.4%)	<0.001
II	587 (35.3%)	279 (39.0%)	249 (36.8%)	59 (21.9%)	
III/IV	1,006 (60.5%)	387 (54.1%)	409 (60.5%)	210 (77.8%)	
HR status					
Negative	553 (33.3%)	149 (20.8%)	232 (34.3%)	172 (63.7%)	<0.001
Positive	1,109 (66.7%)	567 (79.2%)	444 (65.7%)	98 (36.3%)	
HER2 status					
Negative	979 (58.9%)	223 (31.1%)	501 (74.1%)	255 (94.4%)	<0.001
Positive	683 (41.1%)	493 (68.9%)	175 (25.9%)	15 (5.6%)	
Brain metastasis					
No	1,544 (92.9%)	712 (99.4%)	625 (92.5%)	207 (76.7%)	<0.001
Yes	118 (7.1%)	4 (0.6%)	51 (7.5%)	63 (23.3%)	
Lung metastasis					
No	1,106 (66.5%)	597 (83.4%)	413 (61.1%)	96 (35.6%)	<0.001
Yes	556 (33.5%)	119 (16.6%)	263 (38.9%)	174 (64.4%)	
Bone metastasis					
No	708 (42.6%)	392 (54.7%)	234 (34.6%)	82 (30.4%)	<0.001
Yes	954 (57.4%)	324 (45.3%)	442 (65.4%)	188 (69.6%)	
Surgery of primary site					
No	1,107 (66.6%)	447 (62.4%)	470 (69.5%)	190 (70.4%)	0.007
Yes	555 (33.4%)	269 (37.6%)	206 (30.5%)	80 (29.6%)	
Chemotherapy					
No	439 (26.4%)	134 (18.7%)	202 (29.9%)	103 (38.1%)	<0.001
Yes	1,223 (73.6%)	582 (81.3%)	474 (70.1%)	167 (61.9%)	
Radiotherapy					
No	1,409 (84.8%)	593 (82.8%)	587 (86.8%)	229 (84.8%)	0.114
Yes	253 (15.2%)	123 (17.2%)	89 (13.2%)	41 (15.2%)	

HR, hormone receptor; HER2, human epidermal growth factor receptor 2; PI, Pacific Islander; AI, American Indian; AN, Alaska Native; IDC, invasive ductal carcinoma; ILC, invasive lobular carcinoma.

### Prognostic Score and Validation

**Figure 2:**
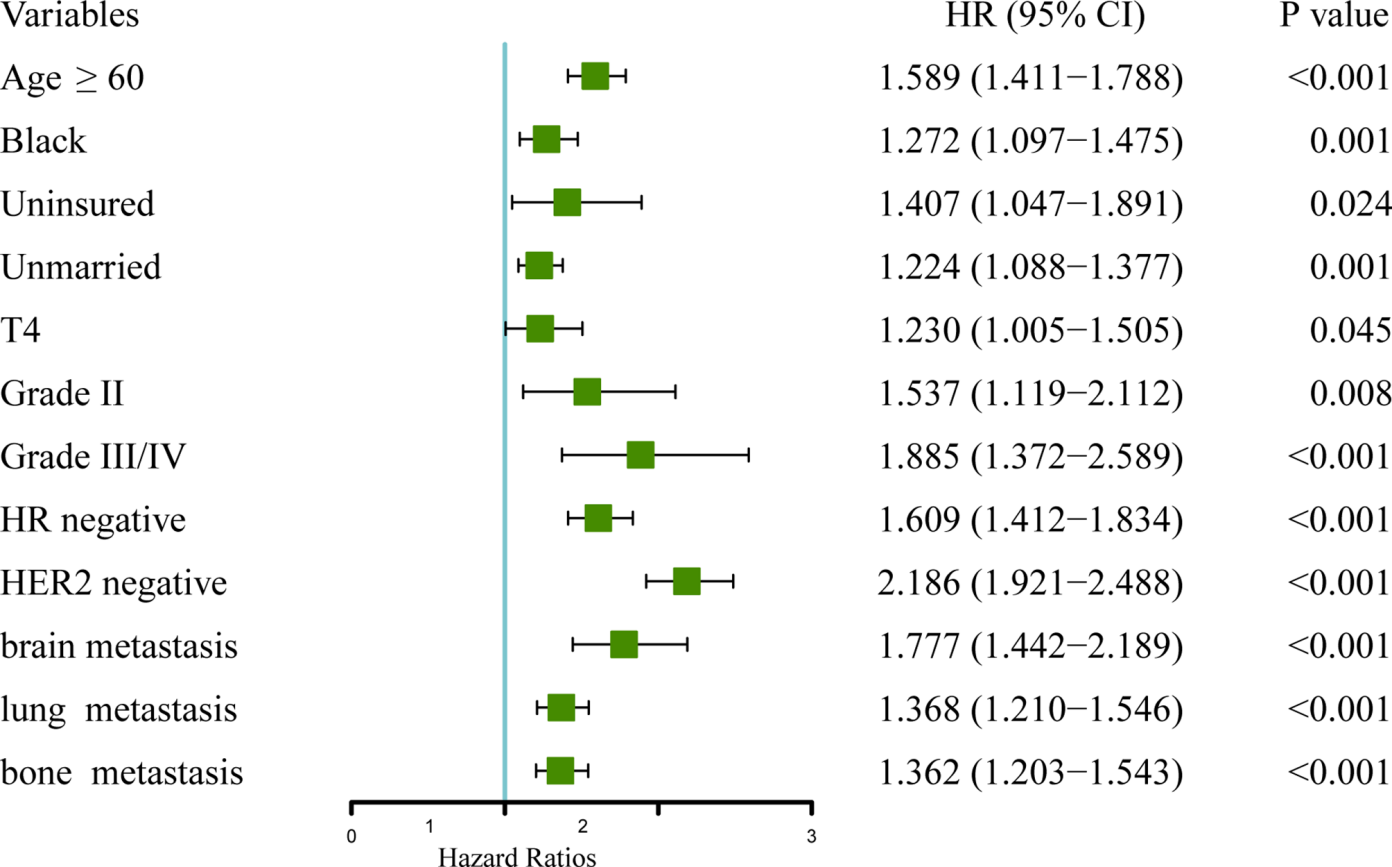
Forest plot showing the results in the multivariate Cox regression analysis.

Multivariable analysis showed that age, race, insurance and marital status, T stage, pathological grade, molecular subtypes (HR and HER2 status), and extrahepatic metastasis (brain, lung, and bone metastases) were independent prognostic factors (**Table 2** and **Figure 2**). Specifically, older age at diagnosis (age ≥60: HR 1.589, 95% CI 1.411–1.788; <60 as a reference) and black (HR 1.272, 95% CI 1.272–1.475; white as a reference) increased the risk of death dramatically. The risk of mortality was also higher in uninsured (HR 1.407, 95% CI 1.047–1.891) or unmarried (HR 1.224, 95% CI 1.088–1.377) patients. There was also a correlation between higher T stage (T4: HR 1.230, 95% CI 1.005–1.505; T1 as a reference) or pathological grade (II: HR 1.537, 95% CI 1.119–2.112; III/IV: HR 1.885, 95% CI 1.372–2.589; I as a reference) and poorer overall survival. Additionally, negative status of HR (HR 1.609, 95% CI 1.412–1.834) and HER2 (HR 2.186, 95% CI 1.921–2.488) and the involvement of extrahepatic metastatic sites, including brain (HR 1.777, 95% CI 1.442–2.189), lung (HR 1.368, 95% CI 1.210–1.546), and bone (HR 1.362, 95% CI 1.203–1.543), were negative prognostic factors for long-term outcome of *de novo* BCLM patients.

**Table 2:** Multivariate Cox regression model of training set.

Variables in the Equation	B	SE	Wald	df	*P* value	Exp(B)	95% CI	
Age	0.463	0.060	58.699	1	0.000	1.589	1.411	1.788
Race			12.852	3	0.005			
Black	0.241	0.075	10.157	1	0.001	1.272	1.097	1.475
PI	−0.131	0.123	1.122	1	0.290	0.878	0.689	1.118
AI	−0.362	0.580	0.388	1	0.533	0.696	0.223	2.172
Insurance status	0.341	0.151	5.119	1	0.024	1.407	1.047	1.891
Marital status	0.202	0.060	11.373	1	0.001	1.224	1.088	1.377
T			12.677	3	0.005			
T2	−0.016	0.104	0.023	1	0.879	0.984	0.803	1.207
T3	−0.017	0.114	0.022	1	0.881	0.983	0.786	1.230
T4	0.207	0.103	4.026	1	0.045	1.230	1.005	1.505
Grade			20.844	2	0.000			
II *vs* I	0.430	0.162	7.050	1	0.008	1.537	1.119	2.112
III/IV	0.634	0.162	15.307	1	0.000	1.885	1.372	2.589
HR Status	0.476	0.067	50.742	1	0.000	1.609	1.412	1.834
HER2 Status	0.782	0.066	140.342	1	0.000	2.186	1.921	2.488
brain	0.575	0.106	29.167	1	0.000	1.777	1.442	2.189
lung	0.313	0.062	25.164	1	0.000	1.368	1.210	1.546
bone	0.309	0.064	23.591	1	0.000	1.362	1.203	1.543

HR, hormone receptor; HER2, human epidermal growth factor receptor 2; PI, Pacific Islander; AI, American Indian; AN, Alaska Native; B, regression coefficient; SE, standard error; Wald, test statistic; df, degrees of freedom; Exp(B), hazard ratio.

**Figure 3:**
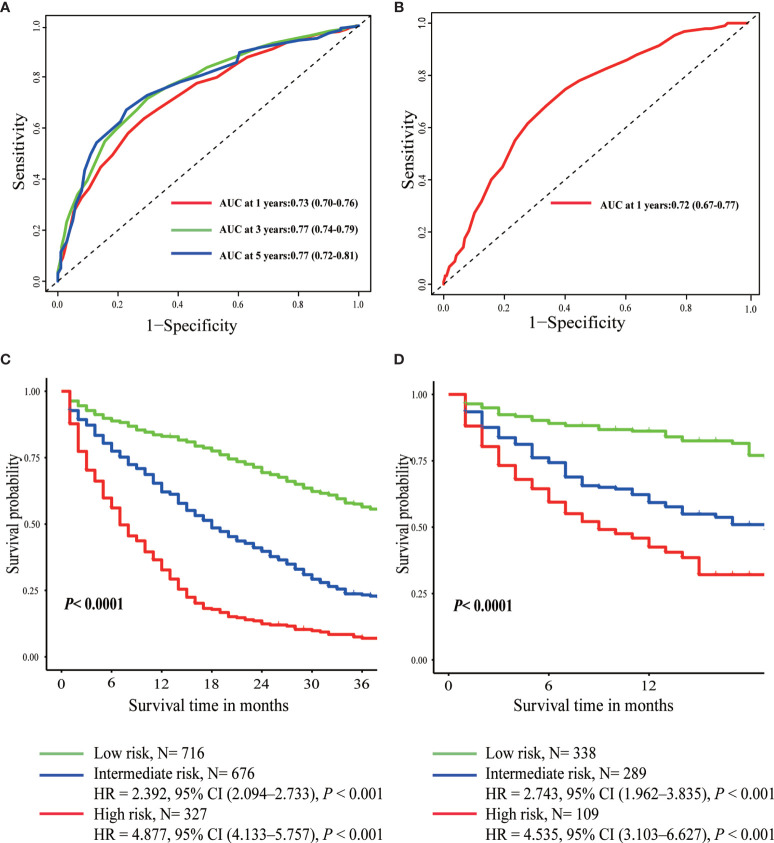
Time-dependent ROC curves of prognostic score in the training set **(A)** and the validation set **(B)**. Overall survival curves plotted by Kaplan-Meier method in the training set **(C)** and the validation set **(D)**.

**Figure 4:**
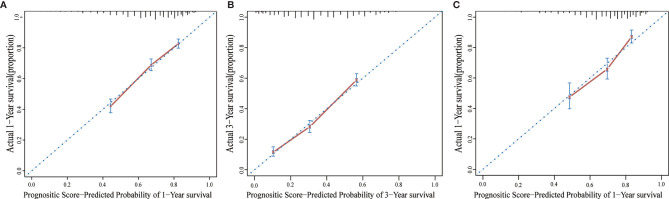
The calibration curve for predicting patient survival at 1 year **(A)** and 3 years **(B)** in the training set and at 1 year **(C)** in the validation set.

Furthermore, scores for each variable were determined based on their regression coefficients in the multivariable Cox model, and therefore overall prognostic score integrating these 11 parameters was constructed (**Table 3**). The discrimination ability of overall prognostic score was assessed by constructing time-dependent ROC curves and calculating the AUC (**Figure 3**). The AUC was 0.77 and 0.72 at 1 year for the training and validation sets, respectively, demonstrating high discrimination power of the prognostic score. Moreover, the calibration plots of the two datasets suggested that the prognostic score prediction had excellent agreement with the actual observation (**Figure 4**).

**Table 3:** Calculation of the score and cut-off points of the risk stratification.

Parameter	Value	Points		
Age	≥60	5		
Race	Black	2		
Insurance status	Uninsured	3		
Marital status	Unmarried	2		
T	4	2		
Grade	II	4		
	III/IV	6		
HR	Negative	5		
HER2	Negative	8		
Brain metastasis	Yes	6		
Lung metastasis	Yes	3		
Bone metastasis	Yes	3		
For all other values		0		
Points	Risk stratification	1-year survival	3-year survival	Median overall survival, months
<18	Low-risk	83%	56%	44 (40–49)
18–25	Intermediate-risk	62%	23%	18 (16–20)
>25	High-risk	33%	7%	7 (7–10)

HR, hormone receptor; HER2, human epidermal growth factor receptor 2.

### Risk Stratification and Subgroup Analysis

According to the optimal cut-off value determined by X-tile software, patients were divided into three different risk groups: low-risk (score <18), intermediate-risk (score 18–25), and high-risk (score >25) group (**Supplementary Figure 1** and **Table 3**). The median OS of the three prognostic groups were 44, 18, and 7 months, with a 3-year survival rate of 56, 23, and 7%, respectively (**Table 3**). As was shown in **Figure 3**, intermediate-risk and high-risk groups demonstrated higher risk of death than low-risk group in both the training (intermediate-risk: HR 2.392, 95% CI 2.094–2.733; high-risk: HR 4.887, 95% CI 4.133–5.757; low-risk as a reference) and validation sets (intermediate-risk: HR 2.743, 95% CI 1.962–3.835; high-risk: HR 4.535, 95% CI 3.103–6.627; low-risk as a reference).

**Figure 5:**
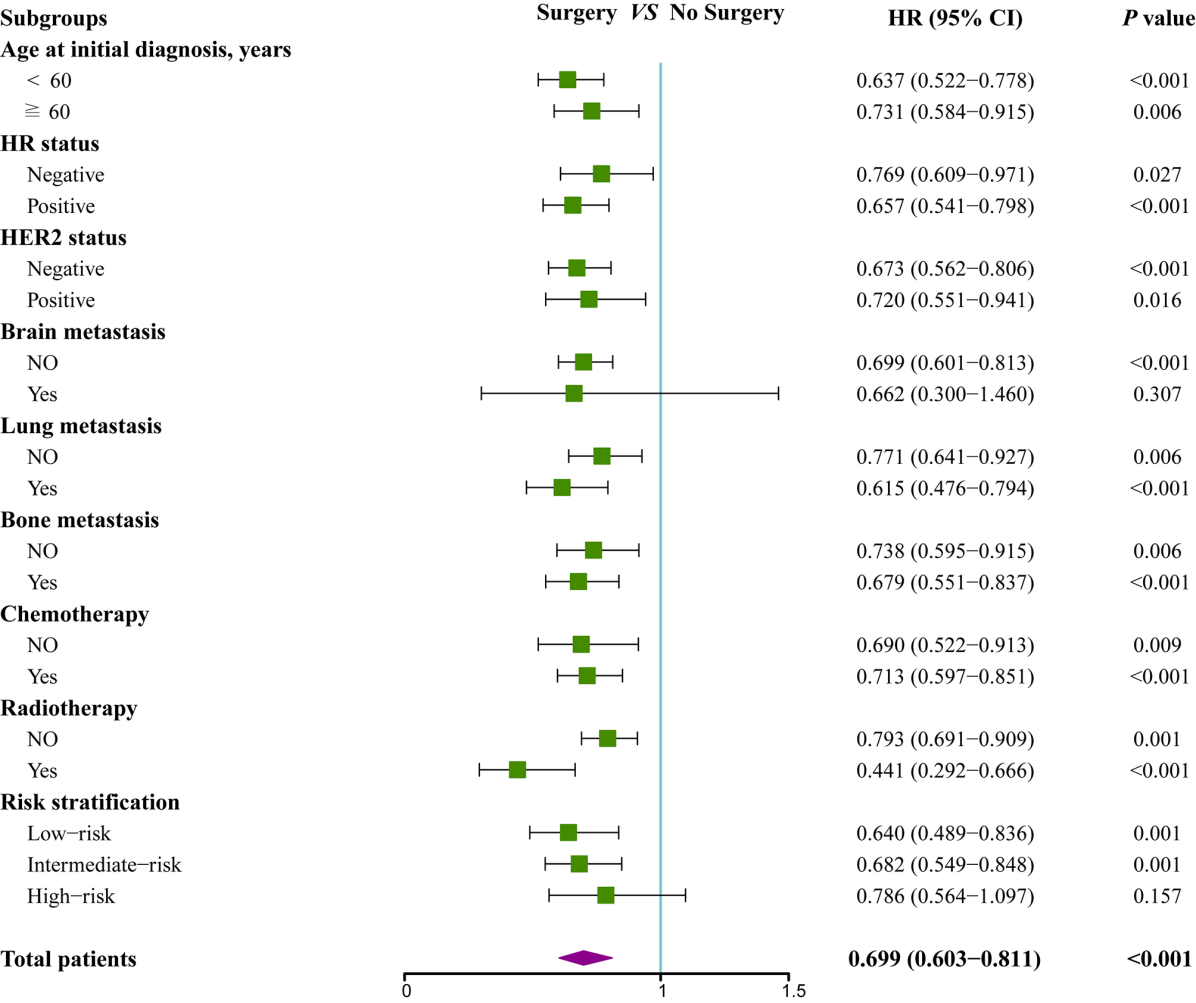
Subgroup analysis of overall survival for patients who received locoregional treatment or not.

**Figure 6:**
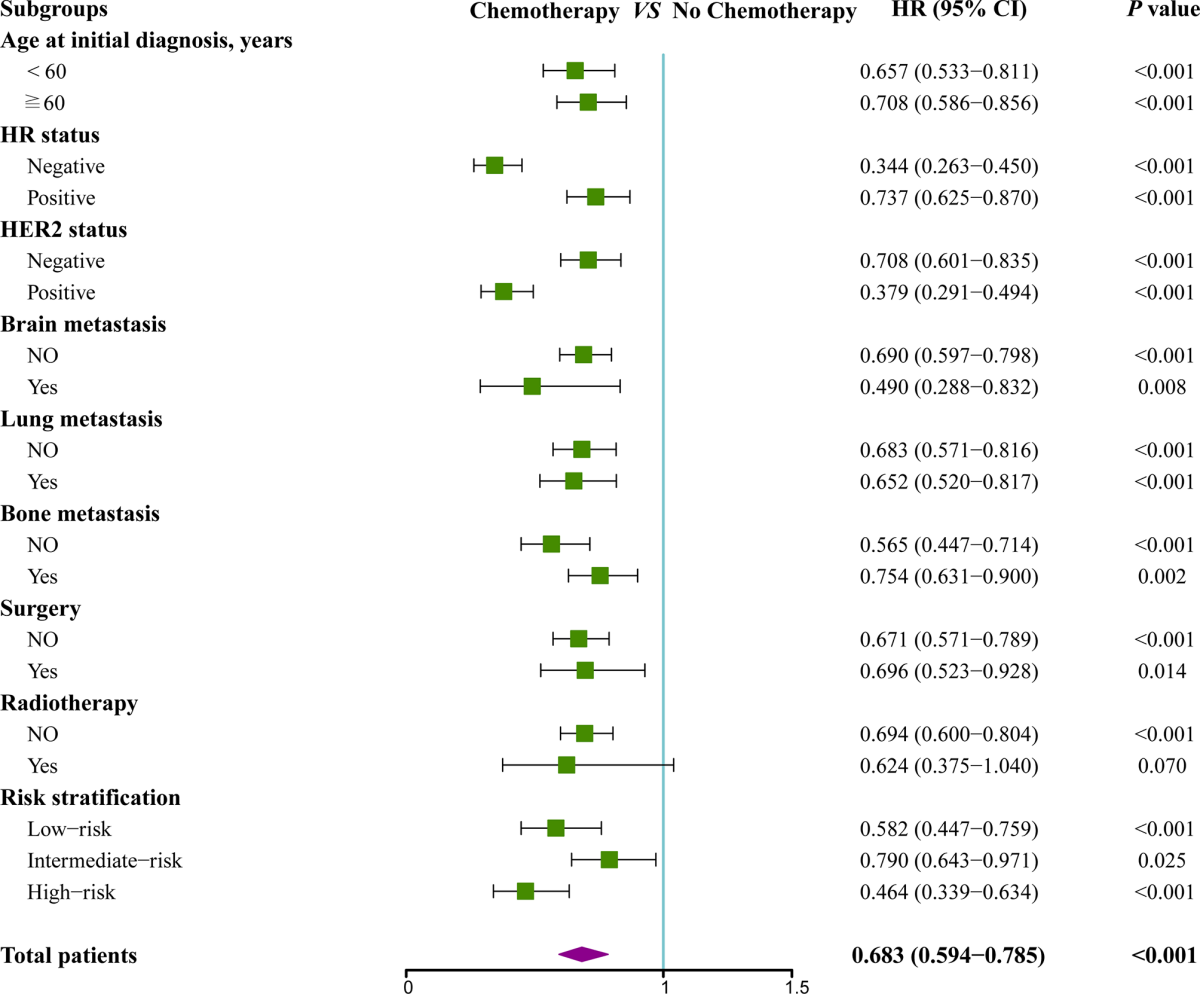
Subgroup analysis of overall survival for patients who received chemotherapy or not.

To further assess whether the prognostic score was also effective for treatment guidance, subgroup analysis was performed to explore surgery of primary site and chemotherapy benefit in different risk groups (**Figures 5** and **6**). The results found that patients in low-risk (HR 0.64; 95% CI 0.49–0.84; *P*=0.001) and intermediate-risk groups (HR 0.68; 95% CI 0.55–0.85; *P*=0.001) could benefit from locoregional surgical treatment, but it seemed not to prolong the survival time of patients of high-risk group (HR 0.79; 95% CI 0.56–1.10; *P*=0.157) significantly (**Figure 5**). However, chemotherapy could produce a significant survival benefit in these three different risk groups (**Figure 6**).

## Discussion

The present study focused on developing a prognostic score for *de novo* BCLM patients to improve prognosis prediction and guide clinical treatment. Our findings indicate that age, race, insurance and marital status, T stage, pathological grade, molecular subtypes, and extrahepatic metastasis were independent predictors of mortality in *de novo* BCLM patients. Therefore, the overall prognostic score including these factors was constructed, which had excellent predictive performance validated by time-dependent ROC curves and calibration plots. Moreover, the risk stratification on the basis of the overall prognostic score could have greater practical value in determining prognosis and making clinical decision.

In our study, advanced age was an independent negative predictor for OS partially due to undertreatment of chemotherapy or radiotherapy in these populations (14). Socioeconomic factors may explain why uninsured or unmarried patients had a higher odds of death (15). Notably, sociodemographic factors, treatment factors, and differentially expressed genes may lead to the survival disparity between black and white patients (16, 17). Higher T stage, higher pathological grade, and multiple extrahepatic metastases represented more aggressive biological behavior and larger tumor burden, resulting in relatively unfavorable prognosis. In terms of molecular subtypes, significantly better overall survival for patients with HR-positive breast cancer may be attributed to safe and effective endocrine therapy, while the advent of several HER2-targeted therapies also has significantly reversed inferior survival outcomes of HER2-positive breast cancer, making the median survival of advanced HER2-positive breast cancer now approach 5 years (18). Although numerous studies have demonstrated the role of tumor characteristics and treatment options in the survival regarding *de novo* BCLM patients, few prognostic models for these patients have been constructed. The present study developed the prognostic score integrating readily obtained variables and the risk stratification to subdivide patients into different risk groups for clinicians to indicate prognosis of *de novo* BCLM patients.

The latest guidelines recommend that systemic therapy remains a cornerstone of treatment for MBC, including BCLM (9, 19). Our study also found that chemotherapy could confer long-term survival benefit for nearly all subgroups. Nevertheless, surgical resection of the primary tumor was also expected to be an effective therapeutic intervention because multiple retrospective analyses reported a survival gain from locoregional treatment in *de novo* MBC patients (20–22). However, prospective clinical trials evaluating its value reached distinctly conflicting conclusions. A Turkish trial (MF07-01, NCT00557986) compared initial locoregional treatment (LRT) plus subsequent systemic therapy with primary systemic therapy alone for *de novo* MBC, reporting a 34% lower risk of death and a 17.2% higher 5-year OS (41.6 *vs* 24.4%) for patients in LRT group, especially in those with younger age, HR-positive and HER2-negative subtypes, or solitary bone-only metastases (23). On the contrary, the Indian study (NCT00193778) suggested that locoregional treatment could not confer overall survival advantage in patients responsive to first-line systemic therapy compared to systemic therapy only (19.2 *vs* 20.5 months), even causing a heavy detriment in distant progression-free survival (11.3 *vs* 19·8 months; HR 1·42, 95% CI 1·08–1·85; *P*=0·012) (24). Another phase III trial (E2108) has drawn a similar conclusion that locoregional treatment following optimal systemic therapy does not improve survival in patients with *de novo* stage IV breast cancer (3-year OS rate, 68.4 *vs.* 67.9%, *P*=0.63) (25). Several significant baseline characteristics including molecular subtypes and post-study treatments such as HER2-targeted therapy were not equally distributed, raising concerns regarding the reliability of trial results (23, 24). In addition, a definitive conclusion on the value of locoregional surgical treatment could not be drawn from current data since different trial designs, study population, and choices of systematic treatments inevitably lead to inconsistency. Therefore, these data suggested that not all *de novo* MBC patients were suitable for locoregional treatment. Similarly, our study revealed that *de novo* BCLM patients in high-risk group or with brain metastasis were not potential candidates for locoregional surgical treatment, indicating that more aggressive tumor biology was closely related with less benefit from primary surgery. Our findings indicated that systemic therapy rather than surgical resection of the primary lesions may be the first choice for *de novo* BCLM patients in high-risk group or with brain metastasis. In addition, clinicians should screen out *de novo* BCLM patients who may benefit from locoregional treatment according to clinical and pathological features with great caution.

There are several limitations in the present study. Firstly, only limited information was available from the SEER database, so other variables could not be included in our research, such as ECOG performance status, Ki-67 expression, liver function parameters, number and size of liver metastasis. Secondly, SEER database could not provide more detailed treatment information, especially timing of surgery, regimens and cycles of chemotherapy, endocrine therapy, and anti-HER2 therapy, to optimize individualized treatment for *de novo* BCLM patients. Additionally, the retrospective research made it difficult to control the potential confounding factors completely to generate more convincing results. As a result of these limitations, further research based on real-world data or prospective clinical trials would be required to determine the best timing to perform local treatment and the specific subgroup benefitting most from it.

## Conclusion

The prognostic score, taking easily obtained clinical variables into account, may facilitate accurate predication of prognosis and individualized treatment guidance. Given the improvement of local disease control resulted from locoregional treatment and long-term survival following new therapeutic strategies including CDK4/6-inhibitors plus endocrine therapy or docetaxel, trastuzumab, and pertuzumab (18, 24–26), surgery of primary site may result in the prolongation of survival for selected patients. Due to the fact that the currently available evidence for *de novo* BCLM patients is limited, treatment strategies should be optimized by a multidisciplinary team on the basis of the general condition and willingness of patients, treatment response, and tumor characteristics. Our subgroup analyses may also have implications for evaluating the value of locoregional surgery, but additional randomized trials are warranted.

## Data Availability Statement

Publicly available datasets were analyzed in this study. This data can be found here: SEER.

## Ethics Statement

Ethical approval was provided by the independent ethics committees of Fudan University Shanghai Cancer Center.

## Author Contributions

LJ initiated the project and collected the data. All authors were responsible for data analysis and wrote the first draft. ZW critically reviewed the manuscript. All authors contributed to the article and approved the submitted version.

## Conflict of Interest

The authors declare that the research was conducted in the absence of any commercial or financial relationships that could be construed as a potential conflict of interest.

## Publisher’s Note

All claims expressed in this article are solely those of the authors and do not necessarily represent those of their affiliated organizations, or those of the publisher, the editors and the reviewers. Any product that may be evaluated in this article, or claim that may be made by its manufacturer, is not guaranteed or endorsed by the publisher.

## Abbreviations

BCLM, breast cancer with liver metastasis; SEER, Surveillance, Epidemiology, and End Results; ROC, receiver operating characteristic; AUC, area under the curve; HR, hazard ratio; CI, confidence interval; OS, overall survival; HR, hormone receptor; HER2, human epidermal growth factor receptor 2; MBC, metastatic breast cancer; BCBM, breast cancer with brain metastasis; NCCN, National Comprehensive Cancer Network; RFA, radiofrequency ablation; TACE, transarterial chemoembolization; LRT, locoregional treatment.
