# Supplementary material for: Prognostic Score for De Novo Metastatic Breast Cancer With Liver Metastasis and Its Predictive Value of Locoregional Treatment Benefit
Source: Front Oncol. 2021 Aug 27;11:651636. doi: 10.3389/fonc.2021.651636 (PMC8432710; doi:10.3389/fonc.2021.651636)
Supplement: Supplementary file 1 [file DataSheet_1.pdf]

# Survival Analysis: Total PS

2020/05/13 19:27:28
allenhearst

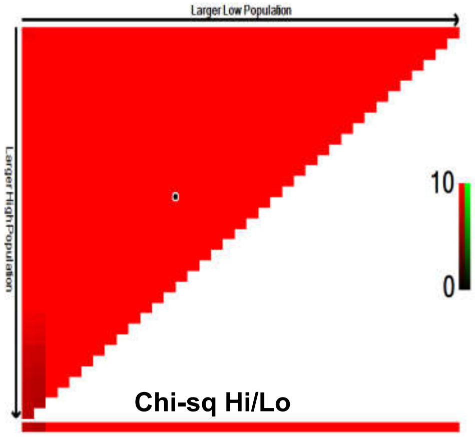


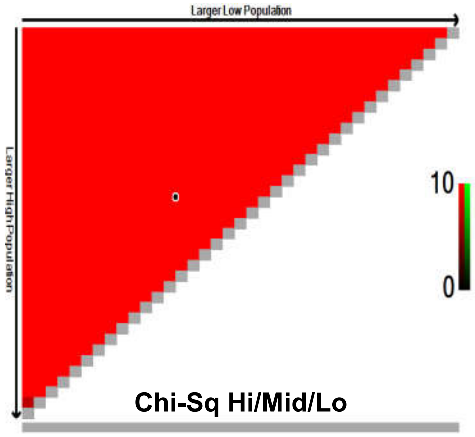


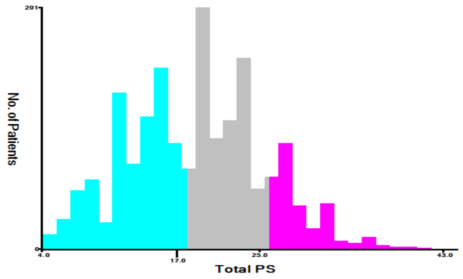


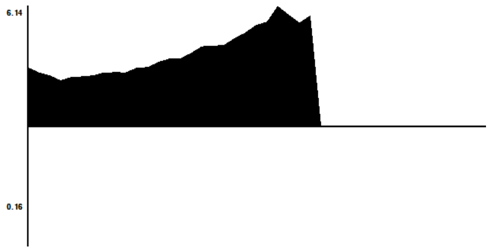


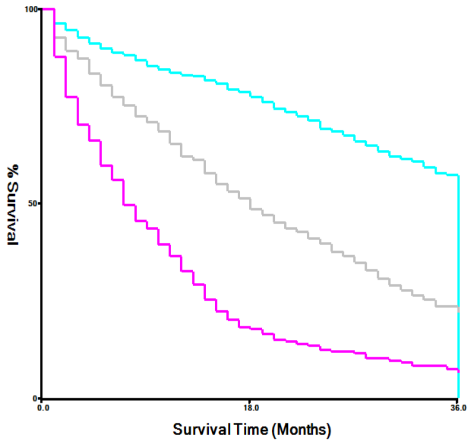


**Subpopulation Cutpoints:**

| Pt No | % Total | Events | Rate | Rank | Range |
|---|---|---|---|---|---|
| 716 | 43.08 | 296 | 41.34 | 0 to 13 | 4.00 thru 17.00 |
| 676 | 40.67 | 501 | 74.11 | 14 to 21 | 18.00 thru 25.00 |
| 270 | 16.25 | 245 | 90.74 | 22 to 37 | 26.00 thru 43.00 |
| 1662 | 100.00 | 1042 | 62.70 | 0 to 37 | 4.00 thru 43.00 |

**Statistics:**

| Variable | Value | |
|---|---|---|
| Chi-Sq Hi/Mid/Lo | 415.8683 | Max: 423.2047 |
| Lo vs Mid | 170.7433 | |
| Mid vs Hi | 97.2780 | |
| Lo vs Hi | 392.9110 | |
| Relative Risk 1 vs 2 vs 3 | 1.00 / 1.79 / 2.19 | |

**Copyright: Camp/Rimm, Yale University**
**www.tissuearray.org**
