# Supplementary material for: Prognostic Score for De Novo Metastatic Breast Cancer With Liver Metastasis and Its Predictive Value of Locoregional Treatment Benefit
Source: Front Oncol. 2021 Aug 27;11:651636. doi: 10.3389/fonc.2021.651636 (PMC8432710; doi:10.3389/fonc.2021.651636)
Supplement: Supplementary file 2 [file Table_1.docx]

| Supplementary Table 1. Characteristics of BCLM and risk stratification in the validation set | | | | | |
| --- | --- | --- | --- | --- | --- |
|  |  | Risk stratification | | | *P* Value |
|  | Total  N=736 (100%) | Low-risk  N=338 (45.9%) | Intermediate-risk  N=289 (39.3%) | High-risk  N=109 (14.8%) |  |
| Age at initial diagnosis, years | | | | | |
| < 60 | 433 (58.8%) | 260 (76.9%) | 140 (48.4%) | 33 (30.3%) | < 0.001 |
| ≥ 60 | 303 (41.2%) | 78 (23.1%) | 149 (51.6%) | 76 (69.7%) |  |
| Gender | | | | | |
| Male | 1 (0.1%) | 0 | 1 (0.3% ) | 0 | 0.541 |
| Female | 735 (99.9%) | 338 (100%) | 288 (99.7%) | 109 (100%) |  |
| Race | | | | | |
| White | 540 (73.4%) | 258 (76.3%) | 215 (74.4%) | 67 (61.5%) | < 0.001 |
| Black | 131 (17.8%) | 40 (11.8%) | 53 (18.3%) | 38 (34.9%) |  |
| Asian or PI | 60 ( 8.2%) | 38 (11.2%) | 19 (6.6% ) | 3 (2.8%) |  |
| AI or AN | 5 (0.7%) | 2 (0.6%) | 2 (0.7%) | 1 (0.9% ) |  |
| Insurance status | | | | | |
| Uninsured | 17 (2.3%) | 7 (2.1%) | 5 (1.7%) | 5 (4.6%) | 0.243 |
| Insured | 719 (97.7%) | 331 (97.9%) | 284 (98.3%) | 104 (95.4%) |  |
| Marital status | | | | | |
| Unmarried | 359 (48.8%) | 127 (37.6%) | 154 (53.3%) | 78 (71.6%) | < 0.001 |
| Married | 377 (51.2%) | 211 (62.4%) | 135 (46.7%) | 31 (28.4%) |  |
| T | | | | | |
| 1 | 86 (11.7%) | 46 (13.6%) | 30 (10.4%) | 10 (9.2%) | < 0.001 |
| 2 | 243 (33.0%) | 145 (42.9%) | 77 (26.6%) | 21 (19.3%) |  |
| 3 | 145 (19.7%) | 72 (21.3%) | 54 (18.7%) | 19 (17.4%) |  |
| 4 | 262 (35.6%) | 75 (22.2%) | 128 (44.3%) | 59 (54.1%) |  |
| N | | | | | |
| 0 or 1 | 508 (69.0%) | 241 (71.3%) | 193 (66.8%) | 74 (67.9%) | 0.632 |
| 2 | 98 (13.3%) | 45 (13.3%) | 40 (13.8%) | 13 (11.9%) |  |
| 3 | 130 (17.7%) | 52 (15.4%) | 56 (19.4%) | 22 (20.2%) |  |
| Histological type | | | | | |
| IDC | 612 (83.2%) | 285 (84.3%) | 229 (79.2%) | 98 (89.9%) | 0.031 |
| ILC | 39 (5.3%) | 18 (5.3%) | 21 (7.3%) | 0 |  |
| Other | 85 (11.5%) | 35 (10.4%) | 39 (13.5%) | 11 (10.1%) |  |
| Pathological grade | | | | | |
| I | 29 (3.9%) | 18 (5.3%) | 10 (3.5%) | 1 (0.9%) | < 0.001 |
| II | 259 (35.2%) | 143 (42.3%) | 105 (36.3%) | 11 (10.1%) |  |
| III/IV | 448 (60.9%) | 177 (52.4%) | 174 (60.2%) | 97 (89.0%) |  |
| HR status | | | | | |
| Negative | 243 (33.0%) | 71 (21.0%) | 100 (34.6%) | 72 (66.1%) | < 0.001 |
| Positive | 493 (67.0%) | 267 (79.0%) | 189 (65.4%) | 37 (33.9%) |  |
| HER2 status | | | | | |
| Negative | 403 (54.8%) | 92 (27.2%) | 208 (72.0%) | 103 (94.5%) | < 0.001 |
| Positive | 333 (45.2%) | 246 (72.8%) | 81 (28.0%) | 6 (5.5%) |  |
| Brain metastasis | | | | | |
| NO | 677 (92.0%) | 336 (99.4%) | 262 (90.7%) | 79 (72.5%) | < 0.001 |
| Yes | 59 (8.0%) | 2 (0.6%) | 27 (9.3%) | 30 (27.5%) |  |
| Lung metastasis | | | | | |
| NO | 496 (67.4%) | 279 (82.5%) | 174 (60.2%) | 43 (39.4%) | < 0.001 |
| Yes | 240 (32.6%) | 59 (17.5%) | 115 (39.8%) | 66 (60.6%) |  |
| Bone metastasis | | | | | |
| NO | 286 (38.9%) | 174 (51.5%) | 86 (29.8%) | 26 (23.9%) | < 0.001 |
| Yes | 450 (61.1%) | 164 (48.5%) | 203 (70.2%) | 83 (76.1%) |  |
| Surgery of primary site | | | | | |
| NO | 552 (75.0%) | 235 (69.5%) | 230 (79.6%) | 87 (79.8%) | 0.007 |
| Yes | 184 (25.0%) | 103 (30.5%) | 59 (20.4%) | 22 (20.2%) |  |
| Chemotherapy | | | | | |
| NO | 152 (20.7%) | 52 (15.4%) | 74 (25.6%) | 26 (23.9%) | 0.005 |
| Yes | 584 (79.3%) | 286 (84.6%) | 215 (74.4%) | 83 (76.1%) |  |
| Radiotherapy | | | | | |
| NO | 635 (86.3%) | 286 (84.6%) | 252 (87.2%) | 97 (89.0%) | 0.433 |
| Yes | 101 (13.7%) | 52 (15.4%) | 37 (12.8%) | 12 (11.0%) |  |

NOTE. HR, hormone receptor; HER2, human epidermal growth factor receptor 2;

PI, Pacific Islander;

AI, American Indian; AN, Alaska Native

IDC, Invasive ductal carcinoma; ILC, Invasive lobular carcinoma
